# A zebrafish high throughput screening system used for *Staphylococcus epidermidis* infection marker discovery

**DOI:** 10.1186/1471-2164-14-255

**Published:** 2013-04-15

**Authors:** Wouter J Veneman, Oliver W Stockhammer, Leonie de Boer, Sebastian A J Zaat, Annemarie H Meijer, Herman P Spaink

**Affiliations:** 1 Institute of Biology, Leiden University, PO Box 9502, Leiden RA, 2300, the Netherlands; 2 Department of Medical Microbiology, Center of Infection and Immunity Amsterdam, (CINIMA), Academic Medical Center, PO Box 22660, Amsterdam, 1100 DD, the Netherlands

**Keywords:** Zebrafish, *Staphylococcus epidermidis*, High throughput screening, Transcriptome analysis, RNA deep sequencing, COPAS, Host pathogen interaction

## Abstract

**Background:**

*Staphylococcus epidermidis* bacteria are a major cause of biomaterial-associated infections in modern medicine. Yet there is little known about the host responses against this normally innocent bacterium in the context of infection of biomaterials. In order to better understand the factors involved in this process, a whole animal model with high throughput screening possibilities and markers for studying the host response to *S. epidermidis* infection are required.

**Results:**

We have used a zebrafish yolk injection system to study bacterial proliferation and the host response in a time course experiment of *S. epidermidis* infection. By combining an automated microinjection system with complex object parametric analysis and sorting (COPAS) technology we have quantified bacterial proliferation. This system was used together with transcriptome analysis at several time points during the infection period. We show that bacterial colony forming unit (CFU) counting can be replaced by high throughput flow-based fluorescence analysis of embryos enabling high throughput readout. Comparison of the host transcriptome response to *S. epidermidis* and *Mycobacterium marinum* infection in the same system showed that *M. marinum* has a far stronger effect on host gene regulation than *S. epidermidis*. However, multiple genes responded differently to *S. epidermidis* infection than to *M. marinum,* including a cell adhesion gene linked to specific infection by staphylococci in mammals.

**Conclusions:**

Our zebrafish embryo infection model allowed (i) quantitative assessment of bacterial proliferation, (ii) identification of zebrafish genes serving as markers for infection with the opportunistic pathogen *S. epidermidis*, and (iii) comparison of the transcriptome response of infection with *S. epidermidis* and with the pathogen *M. marinum*. As a result we have identified markers that can be used to distinguish common and specific responses to *S. epidermidis*. These markers enable the future integration of our high throughput screening technology with functional analyses of immune response genes and immune modulating factors.

## Background

Infections with *Staphylococcus epidermidis* bacteria pose a serious problem associated with the use of biomaterials in modern medicine [1-5]. These bacteria can form biofilms on the surface of inserted biomaterials and persist in the surrounding tissues, where immune functions are disturbed due to the combined presence of a biomaterial and the bacteria [6,7]. In order to better understand the cause of this phenomenon and to assess the propensity of different bacterial strains and biomaterials to alter and trigger the immune response in the host, a whole animal model with high throughput screening possibilities is desired. This will help identifying which factors determine that innocent bacteria become less susceptible to host defence mechanisms or antibiotic treatments when associated with biomaterials.

Mouse and rat models have been used to investigate *S. epidermidis* infection and biomaterial-associated infection processes. However, histological examination of biopsies is time consuming and does not allow following the infection process over time [8-10]. Even with the use of bioluminescence and fluorescence imaging, high challenge doses are required to visualize bacterial colonization and high throughput screening in rodents is not feasible. However, the zebrafish at the embryonal and larval stages is an excellent model for this purpose: it is translucent, fluorescently labelled immune cells and bacteria can be microscopically imaged in real time, and embryos can be obtained in high numbers [11-15]. The responses of many different pathogens such as *Escherichia coli*, *Mycobacterium marinum*, *Salmonella typhimurium*, *Edwardsiella tarda*, *Burkholderia cenocepacia*, and *Staphylococcus aureus* have already been assessed in zebrafish [16-21]. In previous work we successfully performed extensive transcriptome analyses with *M. marinum*, *S. typhimurium*, and *E. tarda* intravenous infection models using custom made Agilent micro-arrays and deep sequencing [19,22-24]. The conventional infection method for zebrafish embryos is injection of pathogens into the caudal vein. However, this method is labour intensive and low throughput. For that reason we have recently developed and validated a high throughput yolk infection model using *M. marinum* with an automated microinjection system [12]. However, in this high throughput model no transcriptome analysis has been performed until now.

In the present study we have developed a high throughput system for quantitating infection with *S. epidermidis* using the automated microinjection system together with complex object parametric analysis and sorting (COPAS) technology. This quantitative high throughput technology has been used to study the transcriptome responses during non-lethal infection progression of *S. epidermidis* over time using micro-arrays and RNA deep sequencing. In order to understand which responses can be linked to defence mechanisms of the zebrafish towards fish pathogens, we have compared the host responses to *S. epidermidis* and to *M. marinum* at a time point when the initial yolk infection has further spread into the embryo’s tissues. The obtained results allowed us to identify a number of genes as markers common for both infection models but also genes that can be used as markers to discriminate between pathogen specific responses.

## Results and discussion

### Pathogenesis of *S. epidermidis* and *S. aureus* in zebrafish embryos

**Figure 1:**
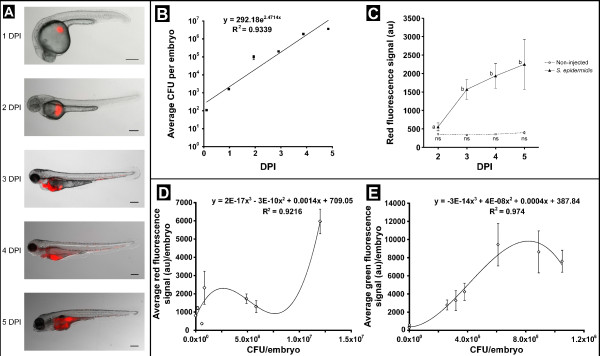
**Quantitation of fluorescence intensity in *S. epidermidis*-injected embryos using the COPAS system.** Panel **A**: Bright field /fluorescence overlay images of mCherry-labelled *S. epidermidis*. Wild type zebrafish embryos injected with 100 CFU of *S. epidermidis* O-47 into the yolk at 2 HPF were imaged at 5 time points from 1 to 5 DPI, scale bar is 250 μm. Panel **B**: CFU counts of *S. epidermidis*-infected embryos. Groups of 10 embryos were homogenized and plated directly after injection until 5 DPI. Panel **C**: The graphs represent the average fluorescence intensity from the entire group of non-injected and *S. epidermidis*-injected embryos, from 2 DPI until 5 DPI. An increase in fluorescence intensity is visible during this infection period. (Error bars = SEM). Different letters indicate statistical significant differences (*P*<0.001). Panels **D** and **E**: Correlation between CFU counts and fluorescence intensity of embryos infected with mCherry-labelled (D) and GFP-labelled (E) bacteria. Pools of 10 infected embryos between 2 and 5 DPI were homogenized and plated. The average fluorescence intensity is plotted against the CFU count.

**Figure 2:**
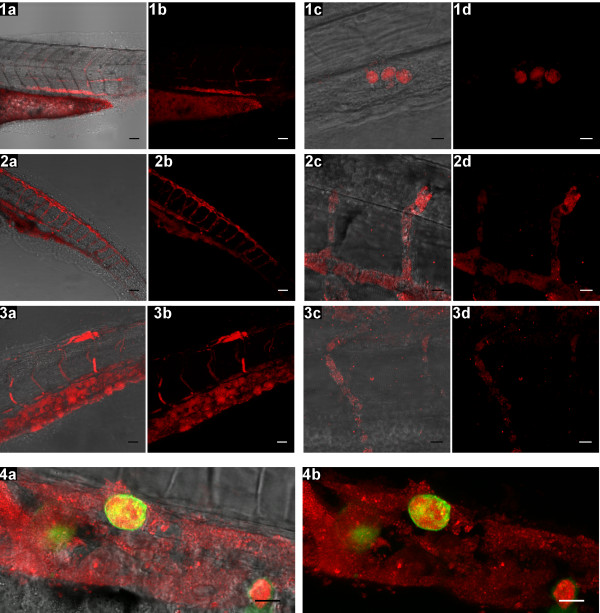
**Invasion of *S. epidermidis *into the zebrafish embryo body.** Confocal z-stacks are shown as transmission/fluorescence overlay (**a** &**c**) and fluorescence images (**b** &**d**). Panel **1**: at 3 DPI mCherry labelled *S. epidermidis* is observed inside the body (**1a** &**1b**, scale bar: 50 μm), and intracellular in the hematopoietic region (**1c** &**1d**, scale bar: 10 μm). Panel **2**: at 4 DPI bacteria are found inside the vasculature (**2a** &**2b**, scale bar: 50 μm), including the intersegmental vessels (**2d** &**2d**, scale bar: 10 μm). Panel **3**: at 5 DPI bacteria are still persisting in the vasculature (**3a** &**3b**, scale bar: 25 μm) and in the intersegmental vessels (**3c** &**3d**, scale bar: 10 μm). Panel **4**: bacteria being taken up by mpeg1:KAEDE positive cells and extracellular in the hematopoietic region at 3 DPI (scale bar: 10 μm).

We first set out to compare *S. epidermidis* infected zebrafish embryos with embryos infected with *S. aureus*. For this purpose we injected *S. epidermidis* O-47 and *S. aureus* RN4220 strains containing GFP or mCherry plasmids under the same conditions into the yolk of embryos at 2 hours post fertilization (HPF). Injections with 5 CFU of *S. aureus* already showed a high intensity of fluorescent bacteria inside the yolk at the first day after injection. At the second day after injection all embryos had died from infection with bacteria spread inside the entire body of the embryos (data not shown). Injection directly into the caudal vein at 28 HPF with approximately 2500 CFU resulted in 100% mortality within several hours (data not shown). This high early mortality due to *S. aureus* is in accordance with earlier reports [20,25,26]. We subsequently tested *S. epidermidis* O-47 in yolk injections at doses of 5, 20, 50 or 100 CFU. At 1 day post injection (DPI) several small spots of fluorescent bacteria were observed inside the yolk with all CFU doses (Figure 1A), which were absent in mock-injected controls. From 2 DPI onwards, bacteria, indicated by their fluorescence signal, were visible inside the yolk in a dose-depended fashion. The fluorescence signal became detectable inside the body of the embryos starting at 3 DPI (Figure 1A). From this day onwards the bacteria were persisting in the vascular system and within various tissues. Confocal laser scanning microscopy was used to obtain a detailed image of bacteria spreading into the different tissues at 3, 4 and 5 DPI, the time points at which spreading of bacteria was observed (Figure 2). At 3 DPI bacteria were found intracellularly and extracellularly in the hematopoietic region (Figure 2, panel 1), and free existing bacteria were observed in the blood were taken up by mpeg1:KAEDE positive cells (Figure 2, panel 4). Free staphylococci in the blood have also been observed after intravascular catheter-related infections [27,28]. At 4 DPI much more extracellular bacteria in the intersegmental vessels were seen (Figure 2, panel 2). No differences were found between the patterns observed at 4 and 5 DPI (Figure 2, panel 3). Although there was strong increase of the bacterial burden in tissues and blood at 4 and 5 DPI (Figure 2 panel 2 & 3 and Additional file 1), in most cases embryos survived the 5 days infection period similar as the mock-injected controls. CFU counts of homogenized pooled embryos revealed that *S. epidermidis* proliferated exponentially inside the embryos during the 5 days of infection (Figure 1B). Comparing the yolk injection method with the traditional caudal vein injection method showed that embryos injected with as much as 5000 and 10000 CFU of *S. epidermidis* into the caudal vein at 28 HPF did not develop any signs of infection. Fluorescence microscopy showed that all injected bacteria were cleared within several hours after injection (data not shown). In view of this, we conclude that the yolk infection system is therefore uniquely suitable to follow the proliferation of *S. epidermidis* and its effects on the host for at least 5 DPI. At the moment we can only speculate why the bacterial that were injected in the yolk had such better survival rates than bacteria injected in the caudal vein at later stages. Three possible explanations (or a combination of these factors) are that (1) there were repeated cycles of invasion from the yolk, (2) the bacteria in the yolk are primed to an infectious growth strategy for instance by using alternate sigma factors [29], or (3) the host immune system has been altered due to the prolonged exposure to high numbers of bacteria and possible associated anti-inflammatory compounds inside the embryos.

### High throughput infection quantification

**Figure 3:**
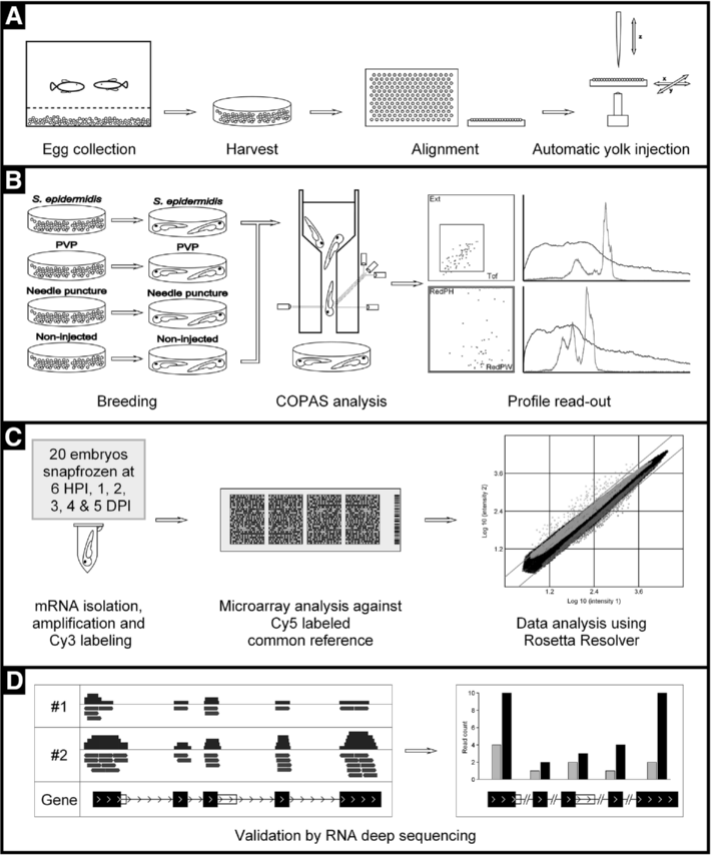
**Workflow of high throughput injection and subsequent analysis.** Panel **A**: from left to right; a zebrafish pair are put together to mate, eggs are collected, eggs are distribute into a 1024 well agarose grid, eggs are injected into the yolk at 2 HPF using the automated microinjection system. Panel **B**: from left to right; after injection, eggs are collected into Petri dishes and incubated at 28°C for a period of 5 days, COPAS analysis is performed on the *S. epidermidis* and non-injected embryos at 2, 3, 4 and 5 DPI. Panel **C**: from left to right; from all groups 20 embryos are snap frozen at 6 HPI, 1, 2, 3, 4 and 5 DPI for RNA isolation, amplification and Cy3 labelling, micro-array analysis against Cy5 labelled common reference and data analysis using Rosetta Resolver. Panel **D**: from left to right; validation of micro-array data was performed by RNAseq analysis of 4 biological replicas of *S. epidermidis* infected embryos at 5 DPI.

The COPAS XL (Union Biometrica, USA) is a large cell flow cytometer designed for fluorescence screening of zebrafish embryos, Drosophila larvae and beads ranging from 1500 to 2000 microns in diameter [12,30]. Samples are analysed for size, optical density and three fluorescence signals. Groups of up to 3000 embryos can simultaneously be analysed and sorted into multi well plates or Petri dishes within 15 minutes (Figure 3). The Profiler software package II detects and analyses up to 8000 data points per object for the extinction and fluorescence channels, and can be used to visualize every sample or to set sorting parameters. The Profiler shows the outline of a sample together with all fluorescence intensity traces, for each of the embryos in a sample. This is exemplified by a typical experiment of *S. epidermidis* infection of zebrafish embryos measured at 4 DPI *(*Figure 3, panel B, and detailed in Additional file 2).

COPAS analysis was performed every day from 2 DPI until 5 DPI. The daily analysis did not cause noticeable damage to the embryos. We observed an increase in the fluorescence signal during the 5 days of infection with *S. epidermidis* (Figure 1C). CFU count results showed good correlation with the increase of fluorescence signal in pools of embryos infected with mCherry-labelled (Figure 1D) or GFP-labelled (Figure 1E) bacteria. However, in the green channel some background signal produced by the embryonic yolk was detected, leading to less accurate quantification (Figure 1E). Since the red fluorescence channel did not show any background signal, the results with mCherry-labelled bacteria were quantitatively more reliable, showing a correlation with the CFU counts (Figure 1D). We did not find any influence of the orientation of the embryos in the flow chamber since we did not detect differences in embryos passing the laser dorsally or ventrally, or with the anterior or posterior side first. Therefore, our results show that combining the COPAS analysis with the automated microinjection system provides a screening system of which the infection levels are statistically reliable.

### Specific marker genes for *S. epidermidis* infection

To characterize the transcriptome response of zebrafish embryos following *S. epidermidis* yolk injection, we performed a time resolved infection experiment using the high throughput set up (Figure 3). Considering that all bacterial injections were carried out with polyvinylpyrrolidone (PVP) as carrier, PVP-injected embryos were taken along to control for possible effects of the carrier. Furthermore, needle puncture treated and non-injected embryo groups were included as additional control groups. Injections were performed with groups of at least 150 embryos of the same parents of which sets of 20 embryos were sampled during 6 time points (Figure 3). In order to check for reproducibility of this experiment an independent experiment was performed with the same parents at 4 DPI. RNA from these samples was used for micro-array analysis using custom made Agilent 4x180k micro-arrays.

**Figure 4:**
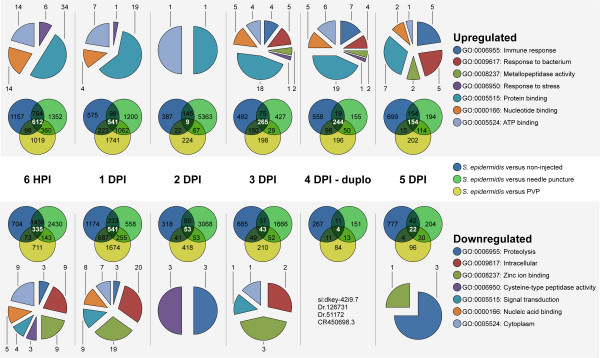
**Overlapping probes in time from micro-array analysis.** The Venn diagrams show the number of significantly up-regulated probes (top row) or down-regulated probes (bottom row) (*P*-value smaller than 10^-8^ and fold changes larger than 2 or smaller than −2) between *S. epidermidis*-injected versus non-injected, *S. epidermidis*-injected versus needle puncture and *S. epidermidis*-injected versus PVP at 6 HPI, 1, 2, 3, 4 and 5 DPI. Data at 4 DPI are based on a biological replica. Pie diagrams represent GO annotation using DAVID bioinformatics resources 6.7 [40,41] from the overlapping genes (in white) at the representative time points.

**Figure 5:**
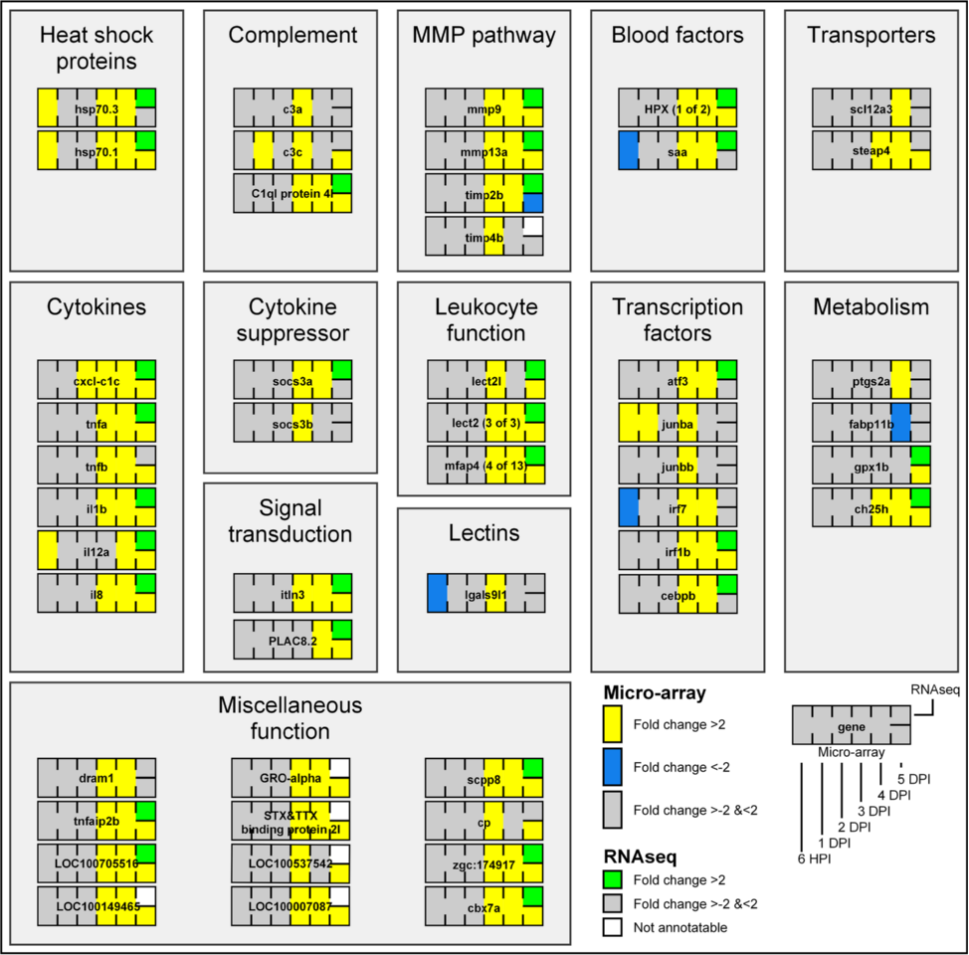
**Gene expression during *S. epidermidis *infection.** Micro-array data are shown of *S. epidermidis*-injected versus non-injected samples at 6 HPI, 1, 2, 3, 4 and 5 DPI time points. The yellow boxes represent up-regulation and the blue boxes down-regulation with a *P*-value smaller than 10^-8^ and fold changes larger than 2 or smaller than −2. The top right bar shows the RNA deep sequencing data, where the green boxes represent significant up regulation. The white boxes could not be identified by RNA deep sequencing. Grey boxes mean that data did not meet the significant criteria. Genes were manually annotated and assigned to functional groups based on GO annotations of the zebrafish genes and their human homologues and on searching on PubMed abstracts. The *D. rerio* Uni-Gene Build # 124 or ENSEMBL Zv9 codes were used as shown with the raw data table in Additional file 4).

Principal component analysis showed a clear signature progression in time of all samples (Additional file 3). Results of statistical analyses are presented in the Venn diagrams of Figure 4. Comparing each time point with multiple control samples clearly shows that there is a false negative effect in the controls that can be corrected for by using the overlap of the ratios of the different controls. This led to a filtered dataset as used for Figure 5 as discussed below. However, we want to emphasize that the injection of PVP has a reproducible effect by itself (Figure 4). This could be of relevance, especially considering the effect of biomaterials on infection capacity of *S. epidermidis* in patients that make it worthwhile to further analyse the effect of PVP on infection in future experiments.

We have analysed the effect of *S. epidermidis* infection over time on gene expression using Unigene clusters and ENSEMBL codes as specified in the raw data table of Additional file 4. Annotation of these probes by Gene Ontology (GO) shows that the most noticeable result is that *S. epidermidis* does induce many immune-related genes starting from 3 DPI, observing a maximum induction of their expression at 4 DPI. Filtering the results (Figure 5) we found an effect on the expression levels of many genes in the earliest measured time point (6 HPI) after exposure to injected bacteria. This effect is diminished to only a few genes whose expression is affected at the time point of 2 DPI, most of which cannot be assigned to a GO category (Figure 4). At 6 HPI, GO analysis indicated very broad classes of gene functions whose expression are affected by infection but did not reveal an obvious link to the immune response since the broad GO category “immune response” was not represented. At this stage the known innate immune responses to bacterial infection are not yet apparent. For instance neutrophils and macrophages have not yet developed and nothing is known about the function of pattern recognition receptors before this stage. We are currently studying the function of the expressed toll-like receptors during early stages of embryogenesis [31]. We have manually annotated various functional categories of genes, of which the transcription levels were strongly affected by infection during time as shown in Figure 5 in a schematic representation and in Additional file 5 in a quantitative manner. Many of the immune genes indicated in Figure 5 have been previously linked to expression in cells of the myeloid lineage in zebrafish [32]. The immune transcriptome response correlates with the infection progression as described above. The first 3 days, the bacteria accumulate inside the embryonic yolk. This apparently does not lead to significantly induction or repression of many immune-related genes. From 3 DPI onwards many immune-related genes were significantly induced with a peak at 4 DPI (Figure 5). At 5 DPI there were slightly less immune-related genes significantly expressed than at 4 DPI. Expression levels of the 49 selected genes shown in Figure 4 at 5 DPI were also lower compared with 4 DPI (Additional file 5). Since the analysed larvae were from the same injected batch it seems that higher microbial burden is not strictly correlated with a stronger immune response.

**Figure 6:**
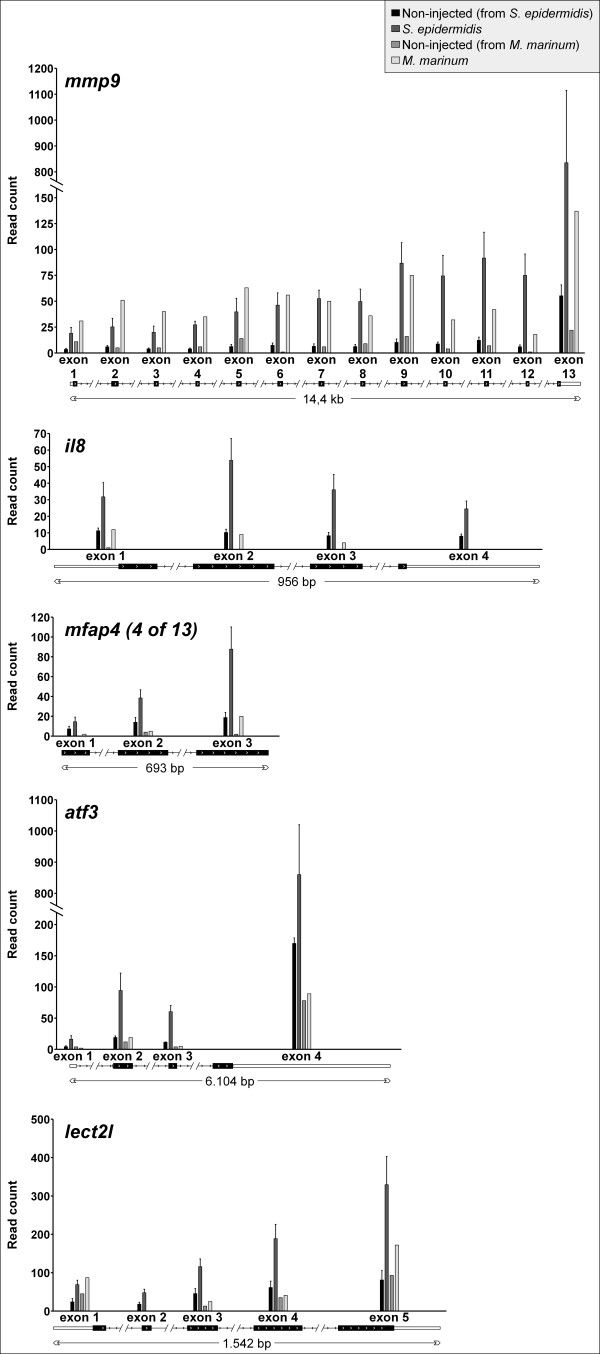
**Expression levels of individual exons.** All exons of *mmp9*, *il8*, *lect2l*, *mfap4* (4 of 13) and *atf3* were significant induced at 5 DPI following yolk infection with *S. epidermidis*. *M. marinum* yolk infection at the same time point only resulted in induction of all *mmp9* exons.

Validation of this micro-array experiment was performed by deep sequencing analysis of RNA samples derived of 4 batches of approximately 150 embryos at the 5 day time point of infection and 4 non-infected batches of embryos. The data confirms the micro-array data as exemplified for some of the most reliable probes (Table 1). Furthermore we have compared the normalized reads per kilobase per million mapped reads (RPKM) [33] for the genes shown in Figure 5. These comparisons show that only in a few cases there are discrepancies between the results of the two technologies. Since the RNA deep sequencing results are obtained with a pool of larger number of biological samples, this could indicate that in these cases the micro-arrays result are less trustworthy. However, it was noted that in cases of discrepancy there were extremely low levels of expression resulting in a very limited number of mapped reads, showing that even with a sequencing depth of at least 20 million reads per sample there is still a limitation of sensitivity of RNA sequencing. This is of note because in most publications currently a sequence depth of 20 million reads is standard for RNA deep sequencing [34,35]. In several cases, such as *il8,* there was no ENSEMBL annotation of the gene that could be used for RPKM analysis. We have manually quantified the number of reads mapping to *il8* to show that there is also induction after infection as in the case of the micro-array analysis (Figure 6). RNA deep sequencing provides a much more detailed insight in gene regulation for instance showing also expression levels for every exon of the genes as shown for the representative genes *mmp9* and *il8* (Figure 6). With the expected progress in development of high throughput bioinformatic pipelines for data visualisation of RNA sequencing data sets, we and others will be able in the near future to further harvest information from our submitted expression datasets as to the effects of infection on differential splicing, transcription start sites or polyadenylation sites in the entire transcriptome.

**Table 1:** Validation of micro-array data by RNA deep sequencing analysis

**Gene**	**Sample**	**Micro-array**		**RNA deep sequencing**	
		**Fold change**	***P*-value**	**Fold change**	***P*-value**
*atf3*	*S. epidermidis* versus non-injected (5DPI)	+ 2.83	2.49E-09	+ 4.77	1.83E-44
*cxcl-c1c*	*S. epidermidis* versus non-injected (5DPI)	+ 5.77	1.77E-14	+ 6.74	4.07E-25
*HPX* (1 of 2)	*S. epidermidis* versus non-injected (5DPI)	+ 10.10	1.60E-11	+ 6.00	1.13E-51
*il1b*	*S. epidermidis* versus non-injected (5DPI)	+ 11.94	1.22E-10	+ 8.54	9.97E-38
*lect2l*	*S. epidermidis* versus non-injected (5DPI)	+ 6.99	1.80E-20	+ 3.12	1.17E-21
*mfap4* (4 of 13)	*S. epidermidis* versus non-injected (5DPI)	+ 21.27	4.15E-41	+ 3.67	4.30E-09
*mmp9*	*S. epidermidis* versus non-injected (5DPI)	+ 5.18	9.73E-20	+ 10.71	1.54E-95
*mmp13a*	*S. epidermidis* versus non-injected (5DPI)	+ 7.35	1.07E-18	+ 15.30	6.03E-82

Shown are 8 representative immune-related genes that were significantly expressed in the micro-array and RNA deep sequencing experiment.

### Comparison of transcriptome responses to *S. epidermidis* and *M. marinum*

**Figure 7:**
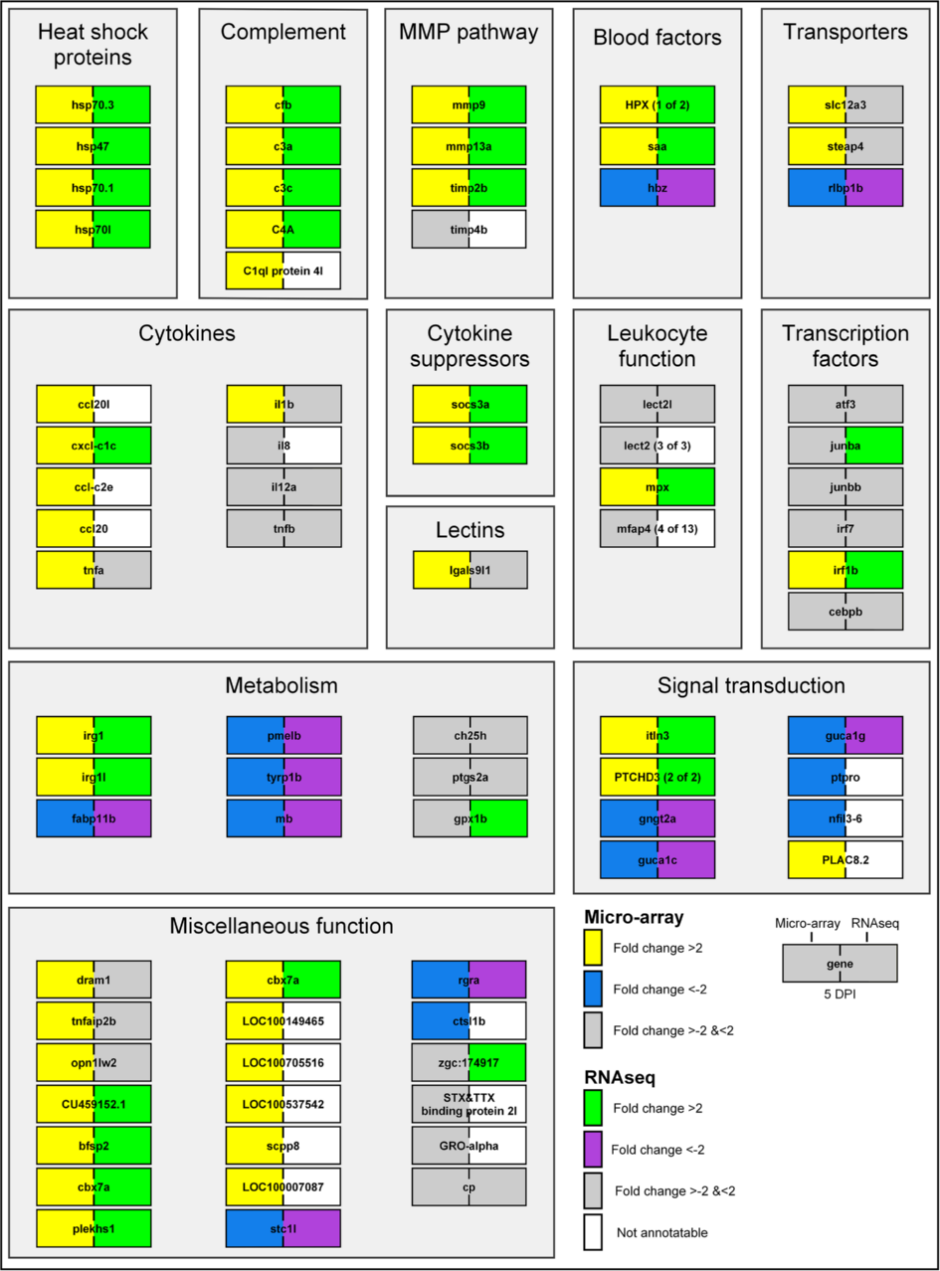
**Gene expression during *M. marinum *E11 infection.** Micro-array data is shown for the infection of *M. marinum* at 5 DPI at the left side of each gene. The yellow boxes represent up-regulation and the blue boxes down-regulation with a *P*-value smaller than 10^-8^ and fold changes larger than 2 or smaller than −2. Probes with unchanged expression are indicated in grey. RNA deep sequencing data is shown for the same sample at the right side of each gene. Green boxes represent up-regulation and the purple boxes down-regulation with a *P*-value smaller than 0.02 and fold changes larger than 2 or smaller than −2. Genes were manually annotated and assigned to functional groups based on GO annotations of the zebrafish genes and their human homologues and on searching on PubMed abstracts. The *D. rerio* Uni-Gene Build # 124 or ENSEMBL Zv9 codes were used as shown with the raw data table in Additional file 6).

In order to compare the transcriptional response observed in zebrafish embryos infected with *S. epidermidis* with the response triggered by a pathogenic bacterium, we also performed an injection experiment with *M. marinum* using the same experimental protocol and sampling at 5 DPI. This time point was chosen in order to make it comparable to previous studies in which the caudal vein was used as the injection site [23]. We performed micro-array analysis, confirming the biological relevance of the yolk injection system since many markers that were previously identified to be differentially expressed in the caudal vein injection system [23] appeared regulated in a similar manner in the high throughput yolk infection system (Figure 7 and Additional file 6). We observed a stronger transcriptional response of immune related genes than what we observed with caudal vein administration, which can be explained by the fact that bacteria accumulated more strongly after five days compared to the caudal vein injection method and have been present one more day inside the embryos.

A number of the immune markers identified to be differentially expressed after infection with *S. epidermidis* appeared regulated in the same way after *M. marinum* yolk infection. These include the matrix metalloproteinases, complement factors, cytokines and heat shock proteins that were previously also identified in *M. marinum* infection in the caudal vein [23]. There are also distinct differences in genes responding to infection by these two different bacteria. Most obvious is a stronger transcriptional response of a number of relevant genes to *M. marinum* than to *S. epidermidis* infection. Interestingly, there is also a category of genes that are highly regulated by *S. epidermis* but not significantly by *M. marinum* in the yolk infection model. These genes include various immune related genes such as *il8*, *il12a*, *tnfb*, *lect2l*, and transcription factor *atf3*, *junba*, *junbb*, *irf7*, *irf1b* and *cebpb*. However, in pilot micro-array studies where higher numbers of mycobacteria were injected, some of the markers were also induced or repressed with the exception of *il12a* and *cebpb* (data not shown). For *M. marinum* infection we observe some difference of gene regulation after yolk infection as compared to caudal vein injection. For instance, *atf3* is upregulated in the latter system (unpublished results). One of the examples of genes induced specifically by *S. epidermidis* in the yolk infection system has high similarity to the mammalian microfibril-associated glycoprotein 4-like isoform 1 gene (*mfap4-*like isoform 1, Unigene accession number Dr.149043, also called *mfap4* (4 of 13) in ENSEMBL) which has been identified previously by Schlosser et al. [36] to bind to human Surfactant protein A (*SP-A*). Interestingly *SP-A* is a good marker for clearance of *S. aureus* since it is involved in binding to the staphylococcal adhesion extracellular adherence protein as well to the macrophage receptors *SP-A* receptor 210 and scavenger receptor class A, enhancing phagocytosis [37]. There are over 13 homologs of this gene clustered in a region on chromosome 1 that are extremely similar but not all inducible by *S. epidermidis* infection. Therefore we aim to further investigate specificity of induction of these genes by microbial infection in follow up studies.

We also performed a RNA sequencing experiment of the *M. marinum* infection system at 5 DPI for verification of the micro-array data. These data show that all exons of *mfap4* (4 of 13), *atf3* and *lect2l* tested are significantly expressed at 5 DPI by *S. epidermidis*, whereas after *M. marinum* infection there is no significant expression of these exons. With *mmp9* as a positive control, the expression of all exons is significantly expressed after *M. marinum* infection (Figure 6)*.*The expression levels for *mfap4* (4 of 13) are based on manual annotation of the unique reads in the known gene region since there are several repetitive DNA regions in common with the other 12 annotated *mfap4* gene family members (Figure 6). Here we clearly benefit from the power of RNA sequencing that can overcome the problems of microarray probe annotation for complex gene families. Therefore using our unbiased approach we were able to confirm known immune genes as markers for staphylococci infection but also identify novel markers as good candidates for specific response markers of *S. epidermidis* in our infection model that we will study further in functional analysis in the near future.

## Conclusions

Microscopic imaging showed that *S. epidermidis* when injected into the yolk or caudal vein proved to be far less virulent than *S. aureus*. Under the same conditions of yolk injection *S. aureus* immediately invades the body of embryo causing 100% mortality within 3 DPI. In contrast, during the five day time period analysed, *S. epidermidis* proliferates efficiently in the entire body of the infected embryos providing an excellent system for analysis of factors that influence bacterial proliferation and virulence. Based on this advantage, we have developed a versatile high throughput analysis system for bacterial proliferation that is much less time consuming than CFU count determinations, and which allows repeated measurements of the same embryos over time. COPAS analysis proved to be accurate to determine the bacterial burden inside embryos at high throughput. With addition of sorting zebrafish embryos into multi well plates, for automated confocal laser scanning microscopy, a medium throughput, high resolution screenings system can be added. We therefore have extended the high-throughput infection methods developed by Carvalho et al. [12] to a quantitative level and showed the applicability for the analysis of proliferation of opportunistic pathogens such as *S. epidermidis*.

Our over time transcriptome analysis results correlate very well with the infection pattern of *S. epidermidis*. The bacteria will grow for the first 2 to 3 days inside the yolk of the embryos, while from 3 and 4 DPI *S. epidermidis* invade the body of the embryo, at which stage a strong response of many immune related genes occurs. We have compared transcriptome response in the same system using *M. marinum*. These comparisons show that *M. marinum* has a far stronger effect on host gene regulation than *S. epidermidis*. However, some genes were identified that specifically responded to *S. epidermidis* and not to *M. marinum* infection including a cell adhesion gene (*mfap4*, ENSEMBL 4 of 13) that can be linked to specific infection by staphylococci in mammals. Vuong et al. [38] and Otto et al. [26] already reported that *S. epidermidis* itself does not seem to have particular specific virulence factors. All known putative virulence factors have origins in the commensal lifestyle of this species. However, the large difference between the outcome of injection of bacteria into the yolk or caudal vein could have been caused by an effect of prolonged growth of the bacteria in the host organism resulting in a higher virulence when the bacteria are release in other tissues. In our future research we aim to use our identified host marker genes to identify new bacterial traits involved in proliferation in host tissues and the factors that determine their expression during time with emphasis on the time points when bacteria get in contact with immune cells.. We are particularly interested in the effect of biomaterials on possible virulence factors that make virulence deviate from the commensal life style. This can help to understand which host mechanisms and genes are involved during biomaterial-associated infections.

## Methods

### Bacterial strains and growth conditions

*S. epidermidis* strain O-47, containing the GFP expression vector pWVW189 or a derived mCherry expression vector (De Boer L. unpublished) and *S. aureus* strain RN4220 pWVW189 (De Boer L. unpublished) from LB (Luria Bertani) agar plates were cultured overnight at 37°C in 25 ml LB medium supplemented with 10 μg/ml chloramphenicol to mid-log stage. *M. marinum* strain E11 was grown as described in Carvalho et al. [12]. Two reaction vials with 1 ml of the culture were centrifuged at 14680 rpm for 1 min. The pellets were combined and washed three times with 1 ml phosphate-buffered saline (PBS). Suspensions were prepared based on the optical density at 600 nm and by plating and CFU determination. The inocula were suspended in 2% polyvinylpyrrolidone40 (PVP_40_, CalBiochem) to 5.0×10^6^, 1.0×10^8^ CFU/ml.

### Zebrafish husbandry

Zebrafish were handled in compliance with animal welfare regulations and maintained according to standard protocols (http://ZFIN.org). Embryos were grown at 28°C in egg water (60 μg/ml Instant ocean sea salt, Sera Marin). The egg water was refreshed every day.

### Experimental design of infection study

Infection experiments were performed using mixed egg clutches from wild type AB×TL or transgenic UAS:KAEDE/MPEG1:GAL4 strain zebrafish [15]. Embryos were staged at 2 HPF by morphological criteria, and 20 CFU of mCherry or GFP expressing *S. epidermidis* O-47 bacteria suspended in 2% PVP_40_ were injected into the yolk. As a control an equal volume of 2% PVP_40_ was likewise injected. Manual injections were controlled using a Leica M50 stereomicroscope together with a FemtoJet microinjector (Eppendorf) and a micromanipulator with pulled micro capillary needles. Automated microinjections were performed as described in Carvalho et al. [12].

### Microscopy

A Leica fluorescence (MZ 16 FA) stereo microscope and Leica TCS SPE confocal microscope were used to take images of zebrafish embryos. Embryos were kept under anaesthesia (0.02% buffered 3-aminobenzoic acid ethyl ester (Tricaine, Sigma) in egg water) during imaging.

### COPAS analysis

Zebrafish embryos were measured alive every 24 hours until 5 DPI with the COPAS XL using the setting as described below. Photo multiplier tubes (PMT) voltage: 650 V for green/red and 0 V for yellow. Optical density threshold signal was set to 975 mV (COPAS value: 50) and the time of flight (TOF) minimum to 320 μs (COPAS value: 800) in order to reduce the influence of debris.

### CFU count

Injected embryos were collected into a 2 ml reaction vial with a sterile 5 mm stainless steel bead and PBS. The reaction vials were vigorously shaken for 30 seconds at 30 revolutions per second in a shaker (Retsch MM301). All suspensions were diluted, plated in duplicate on LB agar supplemented with 10 μg/ml chloramphenicol, and incubated overnight at 37°C. The following day, colonies were counted using a fluorescence stereo microscope (Leica MZ12_5_).

### Micro-array

Seven parent zebrafish couples kept separately from one another for mating the following week, to perform an identical experiment for a biological replicate. Injections were performed from the 16 cells stage onwards at approximately 2 HPF: the first group was injected with 1 nl 2% PVP_40_ solution containing 20 CFU/nl *S. epidermidis* O-47 pWVW189, the second group with 1 nl 2% PVP_40_ solution without bacteria, the third group only received a needle puncture in the yolk, and the last group was a non-injected control group. Groups consisted of approximately 150 embryos. The *S. epidermidis* O-47 pWVW189-injected group and the non-treated embryos were measured at 2, 3, 4 and 5 DPI with the COPAS XL just before snap freezing. At 6 HPI, 1, 2, 3, 4 and 5 DPI, 20 embryos were collected randomly from each group, snap frozen in liquid nitrogen, and stored at −80°C. Embryos were homogenized in 0.5 ml of TRIzol reagent (Invitrogen), and total RNA was extracted according to the manufacturer’s instructions. RNA samples were treated with DNaseI, (Ambion) to remove residual genomic DNA. RNA integrity was analysed by Lab-on-a-chip analysis (Agilent). The average RIN value of the RNA samples was 8.1 with a minimum of 6.7. Per sample, 500 ng total RNA was combined with Spike A and amplified according to the Agilent Two-Color Microarray-Based Gene Expression Analysis guide version 5.5 (G4140-90050, Agilent technologies). For the common reference an equimolar pool of all test samples was made and 500 ng samples were amplified similarly as the test samples with the exception that Spike B was used. Amino-allyl modified nucleotides were incorporated during the aRNA synthesis (2.5 mM rGAC (GE Healthcare), 0.75 mM rUTP (GE Healthcare), 0.75 mM AA-rUTP (TriLink Biotechnologies). Synthesized aRNA was purified with the E.Z.N.A. MicroElute RNA Clean Up Kit (Omega Bio-Tek). The quality was inspected on the BioAnalyzer (Agilent Technologies) with the Agilent RNA 6000 kit (5067–1511, Agilent Technologies). Test samples were labelled with Cy3 and the Reference sample was labelled with Cy5. For Mycobacterium infected embryos a dye swap technical duplicate was performed in which the control was either labelled with Cy3 or Cy5. The overlap of the technical duplicates was used for the output files. Five μg of aRNA was dried down and dissolved in 50 mM carbonate buffer pH 8.5. Individual vials of Cy3/Cy5 from the mono-reactive dye packs (GE Healthcare) were dissolved in 200 μl DMSO. To each sample, 10 μl of the appropriate CyDye dissolved in DMSO was added and the mixture was incubated for 1 h. Reactions were quenched with the addition of 5 μl 4 M hydroxylamine (Sigma-Aldrich). The labelled aRNA was purified with the E.Z.N.A. MicroElute RNA Clean Up Kit. Yields of aRNA and CyDye incorporation were measured on the NanoDrop ND-1000.Each hybridization mixture was made up from 825 ng Test (Cy3) and 825 ng Reference (Cy5) material. Hybridization mixtures were made as described in the Agilent Two-Color Microarray-Based Gene Expression Analysis guide version 5.5 (G4140-90050, Agilent technologies). The samples were loaded onto 4x180k *D. rerio* micro-arrays (Design ID:028233, Agilent Technologies) and hybridized for 17 hours at 65°C. Afterwards the slides were washed and scanned (20 bit, 3 μm resolution) in an ozone-free room with the Agilent G2505C scanner as described in the Agilent Two-Color Microarray-Based Gene Expression Analysis guide version 5.5 (G4140-90050, Agilent technologies). Data was extracted with Feature Extraction (v10.7.3.1, Agilent Technologies) with the GE2_107_Sep09 protocol for two-color Agilent micro-arrays.

Micro-array data was processed using Rosetta Resolver 7.2 (Rosetta Biosoftware). *S. epidermidis* infection groups were compared to the PVP, needle puncture and non-injected control groups using the Rosetta common reference re-ratio experiment pipeline. Significance cut off for the ratios of *S. epidermidis* versus PVP, *S. epidermidis* versus needle puncture and *S. epidermidis* versus non-injected were set at 2 fold change at *P*-value smaller than 10^-8^. Pathway analysis was performed using the Pathvisio software package (http://www.pathvisio.org) [39] with the same significance cut off. The raw micro-array data have been deposited in the NCBI GEO database under accession number GSE42847 and GSE44352. DAVID bioinformatics resources 6.7 [40,41] was used for gene ontology analysis.

### RNA deep sequencing

Validation of micro-array data was performed by RNAseq analysis. Ten parent zebrafish couples were kept separately from one another for mating the following week, to perform an identical experiment for 4 biological replicates. Injections were performed at approximately 2 HPF using the automated microinjection system. At 5 DPI, embryos were collected from the 2 HPF injected and non-injected group, snap frozen in liquid nitrogen, and stored at −80°C for RNA isolation. Twenty CFU of *S. epidermidis* O-47 pWVW189 were injected to obtain 150 embryos per sample. For Mycobacterium infected embryos approximately 1000 embryos were used with 30 CFU injected per embryo. Embryos were homogenized in 1 ml of TRIzol reagent (Invitrogen), and total RNA was extracted according to the manufacturer’s instructions. RNA samples were treated with DNaseI, (Ambion) to remove residual genomic DNA. RNA integrity was analysed by Lab-on-a-chip analysis (Agilent). The average RIN value of the RNA samples was 9.7 with a minimum of 9.5. A total of 3 μg of RNA was used to make RNA-Seq libraries using the Illumina TruSeq RNA Sample Preparation Kit v2 (Illumina Inc., San Diego, USA). In the manufacturer’s instructions two modifications were made. In the adapter ligation step 1 μl instead of 2.5 μl adaptor was used. In the library size selection step the library fragments were isolated with a double Ampure XP purification with a 0.7x beads to library ration. The resulting mRNA-Seq library was sequenced using an Illumina HiSeq2000 instrument according to the manufacturer’s description with a read length of 2 × 50 nucleotides. Image analysis and base calling was done by the Illumina HCS version 1.15.1. Sequence reads were quality trimmed using the quality_trim module in the CLCbio Assembly Cell v4.0.6. Filtered reads were mapped to ENSEMBL transcripts (Zv9_63) using the ref_assemle_short module in the CLCbio Assembly Cell v4.0.6. Accumulation of transcripts to ENSEMBL genes was done by first converting the mapping files to a table with the assembly_table module in the CLCbio Assembly Cell v4.0.6. Secondly, a custom script was used that sums all reads belonging to the same gene. Non-uniquely mapped reads were divided between genes according to their ratio of uniquely mapped reads. Finally, read counts of transcripts belonging to the same gene were summed to obtain count data at ENSEMBL gene level. Fold-change and differential expression significance values were calculated from gene level read counts using the DESeq package (version 1.8.3) available in Bioconductor (version 2.10). DESeq utilizes a negative binomial distribution for modelling read counts [42]. Secondly reads were counted per exon with a python script (Lodder R. unpublished). Sorted sam files were obtained from the raw fastq files through bowtie 2 [43] and samtools [44]. The raw RNAseq data have been deposited in the NCBI GEO database under accession number GSE42847 and GSE44352.

## Competing interests

The authors declare that they have no competing interests.

## Authors’ contributions

WJV designed and performed experiments, and analysed the data. OWS and LDB contributed to the experiments and data analysis. SAJZ, AHM and HPS conceived and supervised the study. WJV and HPS wrote the first version of the manuscript. All authors assisted with editing of the manuscript and approved the final version.

## Supplementary Material

Additional file 1 **Three dimensional projection of *S. epidermidis *infection spread into the body of 5 DPI embryo.** Click here for file

Additional file 2 **Detailed representation of COPAS profiles.** Shown are data of a representative experiment of *S. epidermidis*-injected embryos at 4 DPI within the operating and profiler software of the COPAS. Both profiles shown as examples are located within the dot plots as indicated. Operating parameters are represented as described in materials and methods. Click here for file

Additional file 3 **Principal component analysis.** Data is mathematically transformed from a number of variables in the expression profiles into a number of uncorrelated variables. It combines three different principal components and shows it into a three-dimensional graph. The first principal component has the largest possible variance followed by the other 2 principal components. Principle component analysis shows that there is a larger effect of time (stage of embryonic development) on the gene expression profiles than of the different treatments. This confirms that the stages of treatment are very similar. Click here for file

Additional file 4 **Unigene clusters and ENSEMBL codes as specified in the raw data table for *S. epidermidis *infection.** Click here for file

Additional file 5 **Quantitative overview of expression levels of Figure 5.** All genes stated in Figure 5 are alphabetically sorted and show the expression levels in a quantitative manner. In this table full data base names are used, whereas in Figure 5 in some instance we have abbreviated the gene names. Click here for file

Additional file 6 **Unigene clusters and ENSEMBL codes as specified in the raw data table for *M. marinum *infection.** Click here for file
