# Supplementary figures and images for: A zebrafish high throughput screening system used for Staphylococcus epidermidis infection marker discovery
Source: BMC Genomics. 2013 Apr 15;14:255. doi: 10.1186/1471-2164-14-255 (PMC3638012; doi:10.1186/1471-2164-14-255)

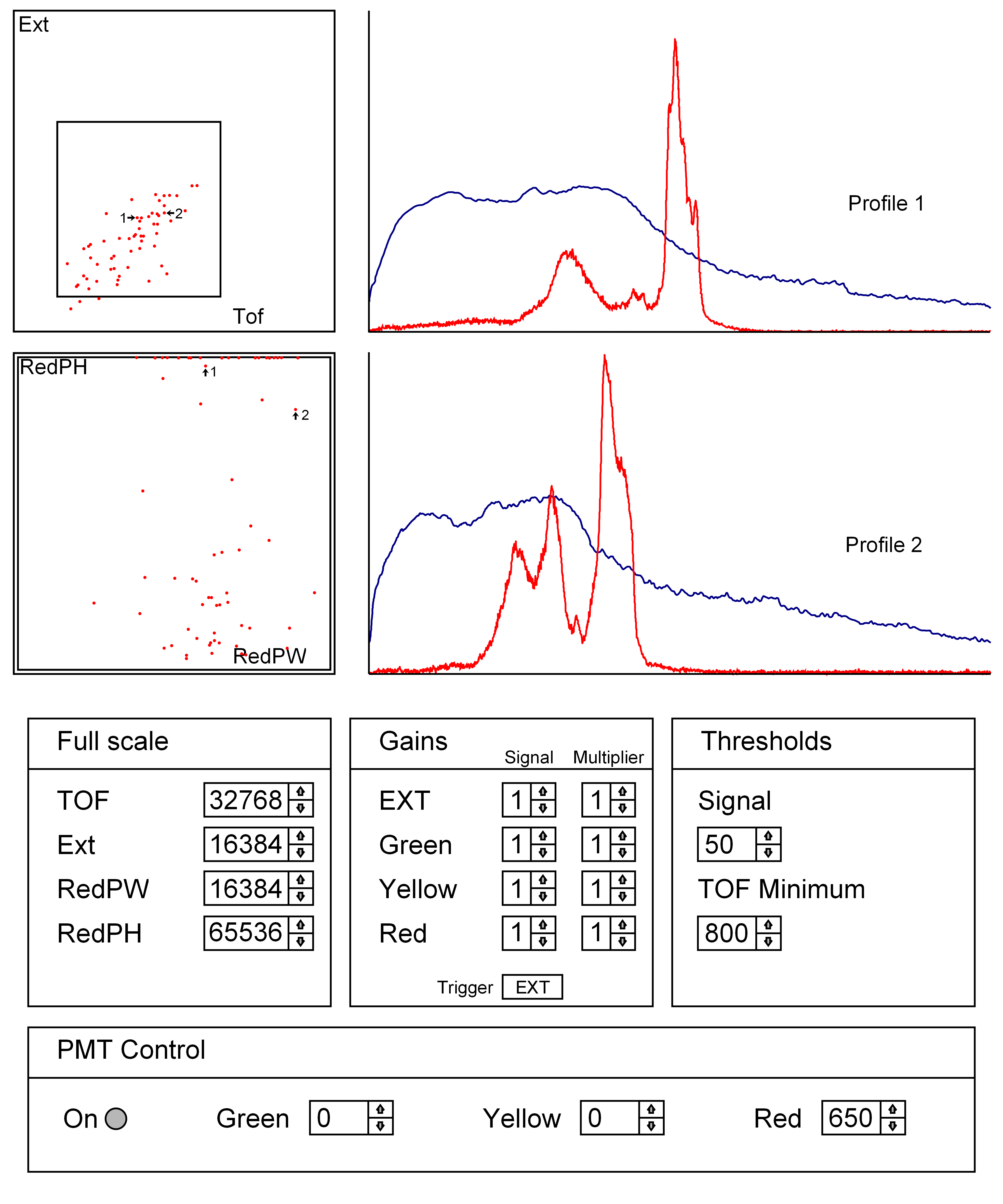

Supplement: Additional file 2 — Detailed representation of COPAS profiles. Shown are data of a representative experiment of S. epidermidis-injected embryos at 4 DPI within the operating and profiler software of the COPAS. Both profiles shown as examples are located within the dot plots as indicated. Operating parameters are represented as described in materials and methods. [file 1471-2164-14-255-S2.png]

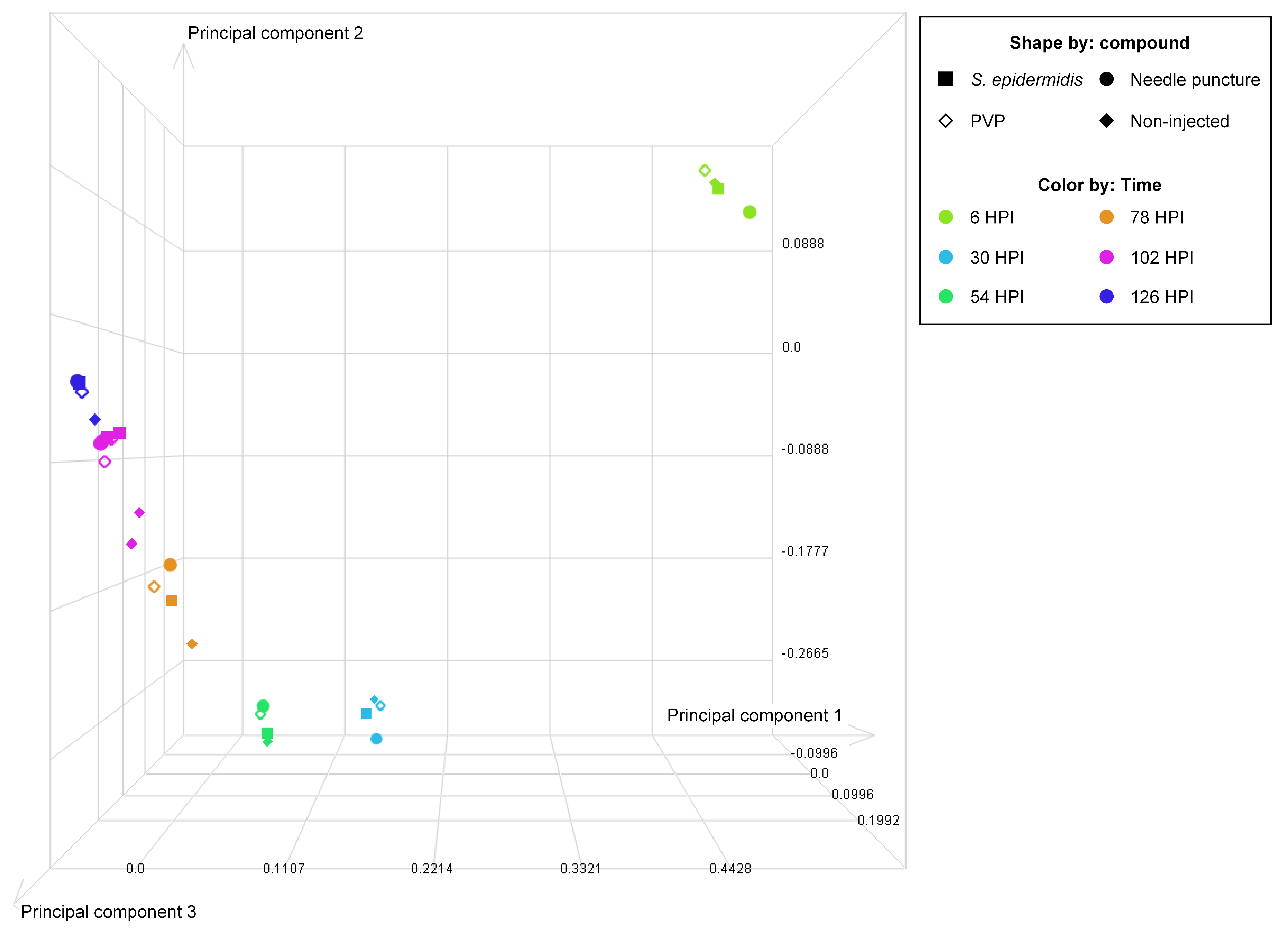

Supplement: Additional file 3 — Principal component analysis. Data is mathematically transformed from a number of variables in the expression profiles into a number of uncorrelated variables. It combines three different principal components and shows it into a three-dimensional graph. The first principal component has the largest possible variance followed by the other 2 principal components. Principle component analysis shows that there is a larger effect of time (stage of embryonic development) on the gene expression profiles than of the different treatments. This confirms that the stages of treatment are very similar. [file 1471-2164-14-255-S3.png]
