# Supplementary material for: A zebrafish high throughput screening system used for Staphylococcus epidermidis infection marker discovery
Source: BMC Genomics. 2013 Apr 15;14:255. doi: 10.1186/1471-2164-14-255 (PMC3638012; doi:10.1186/1471-2164-14-255)
Supplement: Additional file 5 — Quantitative overview of expression levels of Figure 5. All genes stated in Figure 5 are alphabetically sorted and show the expression levels in a quantitative manner. In this table full data base names are used, whereas in Figure 5 in some instance we have abbreviated the gene names. [file 1471-2164-14-255-S5.pdf]

| Gene name | Fold change | | | | | |
|---|---|---|---|---|---|---|
| | 6 HPI | 1 DPI | 2 DPI | 3 DPI | 4 DPI | 5 DPI |
| activating transcription factor 3 (*atf3* ) | | | | 5.6 | 5.6 | 2.8 |
| CCAAT/enhancer binding protein (C/EBP), beta (*cebpb* ) | | | | 4.7 | 3.8 | |
| ceruloplasmin (*cp* ) | | | | 3.2 | | 3.2 |
| chemokine (C-X-C motif) ligand C1c (*cxcl-c1c* ) | | | 7.9 | 8.2 | 14.5 | 5.8 |
| cholesterol 25-hydroxylase (*ch25h* ) | | | | 5.2 | 6.7 | 3.4 |
| chromobox homolog 7a (*cbx7a* ) | | | | 12.8 | 10.3 | 5.3 |
| complement component c3a (*c3a* ) | | | | 5.0 | | |
| complement component c3c (*c3c* ) | | 3.5 | | 3.7 | | 7.3 |
| DNA-damage regulated autophagy modulator 1 (*dram1* ) | | | | 2.9 | 3.0 | |
| fatty acid binding protein 11b (*fabp11b* ) | | | | | -5.3 | |
| glutathione peroxidase 1b (*gpx1b* ) | | | | | | 3.1 |
| heat shock cognate 70-kd protein, tandem duplicate 1 (*hsp70.1* ) | 8.9 | | | 2.9 | 5.1 | 8.1 |
| heat shock cognate 70-kd protein, tandem duplicate 3 (*hsp70.3* ) | 15.6 | | | 2.6 | 4.6 | |
| hemopexin (*HPX* (1 of 2)) | | | | 4.6 | 11.9 | 10.1 |
| intelectin 3 (*itln3* ) | | | | 10.1 | 38.2 | 19.5 |
| interferon regulatory factor 1b (*irf1b* ) | | | | 4.2 | 4.9 | 3.0 |
| interferon regulatory factor 7 (*irf7* ) | -8.3 | | | 5.8 | 4.2 | |
| interleukin 1, beta (*il1b* ) | | | | 5.8 | 11.6 | 11.9 |
| interleukin 12a (*il12a* ) | 6.1 | | | | 3.9 | 4.0 |
| interleukin 8 (*il8* ) | | | | 3.8 | 5.9 | 3.7 |
| jun B proto-oncogene a (*junba* ) | 3.1 | 2.5 | | 2.5 | | |
| jun B proto-oncogene b (*junbb* ) | | | | 3.0 | | |
| lectin, galactoside-binding, soluble, 9 (galectin 9)-like 1 (*lgals9l1* ) | -3.4 | | | | 2.8 | |
| leukocyte cell-derived chemotaxin 2 (*lect2* (3 of 3)) | | | | 8.7 | 14.2 | 7.3 |
| leukocyte cell-derived chemotaxin 2 like (*lect2l* ) | | | | 9.5 | 13.5 | 7.0 |
| matrix metalloproteinase 13a (*mmp13a* ) | | | | 7.3 | 36.9 | 7.4 |
| matrix metalloproteinase 9 (*mmp9* ) | | | | 20.7 | 31.5 | 5.2 |
| placenta-specific 8.2 (*plac8.2* ) | | | | | 32.1 | 318.2 |
| Predicted: complement component 1, q protein 4l ( *C1ql* protein 4l) | | | | 5.3 | 8.9 | 8.4 |
| Predicted: growth regulated oncogene-alpha (*GRO-alpha* ) | | | | 5.2 | 14.4 | 4.7 |
| Predicted: LOC100149465 | | | | 3.6 | 11.6 | 8.2 |
| Predicted: LOC100537542 | | | | 4.5 | | 3.8 |
| Predicted: LOC100705516 | | | | 21.4 | 20.1 | 6.6 |
| Predicted: microfibrillar-associated protein 4 (*MFAP4* (4 of 13)) | | | | 11.5 | 23.7 | 21.3 |
| Predicted: saxitoxin & tetrodotoxin binding protein 2l (*STX* & *TTX* binding protein 2l) | | | | 8.1 | 8.0 | 9.8 |
| prostaglandin-endoperoxide synthase 2a (*ptgs2a* ) | | | | | 2.2 | |
| RIKEN cDNA 9130211l03-like | | | | 7.3 | 6.1 | 4.7 |

| | | | | | | |
|---|---|---|---|---|---|---|
| secretory calcium-binding phosphoprotein 8 (*scpp8* ) | | | | 6.3 | 33.3 | 14.8 |
| serum amyloid A (*saa* ) | -8.2 | | | 65.1 | 51.8 | 29.3 |
| solute carrier family 12 (sodium/chloride transporters), member 3 (*slc12a3* ) | | | | | 2.7 | |
| STEAP family member 4 (*steap4* ) | | | | 2.7 | 2.8 | 2.8 |
| suppressor of cytokine signaling 3a (*socs3a* ) | | | | 4.3 | 2.5 | |
| suppressor of cytokine signaling 3b (*socs3b* ) | | | | 3.4 | | |
| tissue inhibitor of metalloproteinase 2b (*timp2b* ) | | | | 4.2 | 9.6 | -2.5 |
| tissue inhibitor of metalloproteinase 4b (*timp4b* ) | | | | 13.5 | 11.8 | 3.8 |
| tumor necrosis factor a (*tnfa* ) | | | | 35.6 | 13.6 | 12.3 |
| tumor necrosis factor b (*tnfb* ) | | | | 3.3 | 3.8 | 3.6 |
| tumor necrosis factor, alpha-induced protein 2b (*tnfaip2b* ) | | | | 7.7 | 6.9 | 3.1 |
| zgc:174917 | | | | 4.2 | 11.4 | 5.0 |
